# The effect of economic crisis and austerity measures on deaths of despair in Brazil: An interrupted time series analysis

**DOI:** 10.1371/journal.pone.0314294

**Published:** 2024-12-31

**Authors:** Raphael Mendonça Guimarães, Karina Cardoso Meira, Cristiane Teixeira da Silva Vicente, Silvania Suely de Araújo Caribé, Luise Bernardes da Silva Neves, Nicole Almeida Vardiero

**Affiliations:** 1 Oswaldo Cruz Foundation, National School of Public Health, Graduate Program in Public Health, Rio de Janeiro, RJ, Brazil; 2 IDOMED—Estácio de Sá University, School of Medicine, Rio de Janeiro, RJ, Brazil; 3 Federal University of São Paulo, School of Nursing, São Paulo, SP, Brazil; 4 Ministry of Health, Brasília, DF, Brazil; Federal University of Minas Gerais, Nursing School, BRAZIL

## Abstract

Some recent analyses have described that, in the context of the instability of social protection institutions and economic crisis, there is an increase in mortality rates selectively from drug overdoses, suicides and alcohol-related liver diseases. This group of causes was named "Deaths of Despair. In the last decade, Brazil experienced economic stagnation and fiscal austerity, influencing the profile of illnesses and deaths. Therefore, our study aimed to evaluate the effect of the economic crisis and fiscal austerity measures on deaths of despair in Brazil and to describe the trend of deaths of despair in Brazil between 2003 and 2018, according to the phases of the economic cycle. We analyzed the time series of rates by covariates and fitted an interrupted time series model to assess the effect of the crisis on the trend through the Prais-Winsten method. The temporal analysis showed a significant difference in the mean values ​ before and after economic stagnation (Mean 8.68 ± 0.71) and after (Mean 11.62 ± 0.62). We found a positive association between the economic crisis and deaths of despair, with a significant change in level (p-value = 0.003) and a non-significant trend effect (p-value = 0.300). There are differences in sex, age, and especially race: men, middle-aged and black/brown people are more at risk. The present study presents the effect of the economic crisis and mortality in the population, with demographic differences.

## Introduction

The worldwide economy fluctuates in cycles of expansion and downturn with varying durations between them [1]. Nevertheless, relatively stable financial cycles can be somewhat unpredictably interrupted by periods of crisis [2]. The crisis can stem from the bursting of economic bubbles, bank failures, sudden decline in the stock market or other financial products, currency crises, inability to repay debts by national governments, or changes in the political and social scenarios of the countries [3]. Since 2008, the global economy has undergone a severe crisis in the capitalist system [4]. The consequences of this crisis are noticeable in many aspects of life, from the financial market to public health [5,6].

There is very consistent evidence obtained since then that unequivocally links a shift in the mortality pattern with this phase of economic crisis and with the measures to alleviate it, which include fiscal austerity strategies [7–10]. Economic stagnation associated with deindustrialization can lead to a loss of social bonds and individualism and a weakening of workers’ capacity to organize, contributing to social alienation and an increase in all-cause or cause-specific mortality associated with economic crises [9–11]. Psychological factors, such as heightened levels of stress and depression, are crucial indirect causes of excess mortality observed in these periods, possibly due to the uncertainty of the future, as well as the need to adapt to numerous changes in life, including occupational aspects [12]. Furthermore, the economic crisis follows social structure changes and social security dissolution, thereby contributing to increased psychological stress and alterations in the consumption pattern of families and the populace [13].

Recent rises in premature mortality have coincided with decades of economic downturn for less educated and unskilled workers, declining household incomes and marriage rates, increased single-parent families, labor force breakdown, and community decline [11]. Between 1999 and 2013, all-cause mortality for middle-aged, non-Hispanic white men and women in the United States increased by approximately 22% [14]. Also, some recent analyses have depicted a differential increase in mortality rates among middle-aged whites, particularly those with lower educational levels. This rise was primarily attributed to deaths related to drug and alcohol overdoses, suicide, and chronic liver diseases. Case and Deaton [14], followed by Stein et al. [15] and Diez Roux [16]. Consolidate the term "deaths of despair" to describe this group.

Brazil underwent an economic crisis in the last decade. In 2005, the government commenced promoting the recovery of public investments and initiated a phase of fiscal expansion, which persisted with the succeeding government, spanning the period between 2005–2014 [17]. The recovery in public investment mirrored a change in the government’s stance. From there, the government resumed planning and formulating strategic programs and large investment projects. In addition to the increase in public investments, the primary explanation for expanding primary spending was social spending with the increase in social inclusion policies. This dynamism endured until 2010. Since the onset of economic stagnation in Brazil in 2011, because of the global financial crisis that commenced in 2008, with a reduction in the price of commodities, Brazil has encountered one of the most severe periods of recession in its recent history [18]. Between 2011 and 2014, public investments were stagnant, and the proposed fiscal strategy in 2015 was guided by the hypothesis of expansionist austerity [19]. The results fell short of expectations, and the country embarked on a path challenging to resolve in the short term. In 2011, Brazil underwent a rapid economic and fiscal deterioration culminating in the impeachment of then-President Dilma Rousseff. The political coup in 2016 definitively established a recession trend starting in 2015. During this period, there was a reduction in the fiscal adjustment of State investments in health, education, and social security. Santos and Vieira [20] argue that this scenario has contributed to increased unemployment, reduced access to health services, increased prevalence of anxiety and depression, and deaths from suicide, especially in the population aged less than 65 years.

Due to that, there is some concern about the effect of the crisis on health outcomes. However, there is still not enough robust evidence to point to the magnitude of the impact. Therefore, the state of the art indicates a temporal relationship between the intensification of economic tensions and mortality from certain specific causes [14–16]. In this way, this paper has two objects: the first one, to evaluate the effect of the economic crisis and fiscal austerity measures on deaths of despair in Brazil, and the second one, to describe the trend of deaths of despair in Brazil between 2003 and 2018, according to the phases of the economic cycle.

## Methods

### Study design

It is an analytic ecological study of interrupted time series.

### Settings and participants

We evaluated data from Brazil as a whole. For that, we consider the entire population from 2003 to 2018, disaggregated by age group, sex, and race, and obtain the populations for the inter-census years 2001 to 2009 by interpolation. Then, with the estimate of the geometric growth rate from the previous period, we calculate the population estimates from 2011 to 2018.

### Data source

in,providing The data came from two sources: for deaths, we considered information from the Mortality Information System (SIM) by age, sex, race, and cause of death. For the population, we used data from the Brazilian Institute of Geography and Statistics (IBGE) based on population estimates.

Mortality data were extracted from the Mortality Information System (SIM), a comprehensive national database managed by the Brazilian Ministry of Health. SIM systematically collects death records across Brazil, ensuring standardized documentation of mortality events. This database is critical for understanding mortality trends and is continually updated to reflect recent data. For this study, we accessed SIM data to obtain the annual deaths by specific causes relevant to the research objectives, ensuring the most current and complete records.

The Brazilian Institute of Geography and Statistics (IBGE) sourced population estimates and census data. Population counts were based on two decennial Census surveys (2000 and 2010) conducted by IBGE, which provide detailed demographic information, including age, gender, and geographical distribution across Brazilian states and municipalities. For years between these censuses, IBGE generates annual population estimates based on demographic projections, which account for birth, death, and migration rates. These population projections allow for accurate estimation of population trends and calculation of age-standardized mortality rates, which are crucial for analyzing temporal trends across various demographic groups. The methodology employed by IBGE to estimate population counts for non-census years follows statistical models grounded in national demographic patterns, ensuring consistency and reliability across annual estimates.

### Dependent variable

We used the codes of deaths of despair considering the criteria established by Case and Deaton [14,21]. We performed the categorization of causes of death using the codes of the International Classification of Diseases, 10^th^ revision (ICD-10), considering groups as follows: Accidental or intentional poisoning and poisoning of undetermined intent from drug exposure; drugs in the blood; Drug-induced illnesses; Mental/behavioral disorders due to drugs; Alcohol-induced illnesses; Mental/behavioral disorders due to alcohol; Accidental or intentional poisoning and poisoning of undetermined intent form alcohol exposure; Suicide (Table 1).

**Table 1:** Causes of death and ICD-10 codes related to deaths of despair.

Group of causes	ICD-10 codes
Accidental or intentional poisoning and poisoning of undetermined intent from drug exposure; drugs in the blood	X40-X44, X60-64, Y10-Y14; R78.1-R78.5
Drug-induced illnesses	D52.1, D59.0, D59.2, D61.1, D64.2, E06.4, E16.0, E23.1, E24.2, E27.3, E66.1, G21 .1, G24.0, G25.1, G25.4, G25.6, G44.4, G62.0, G72.0, I95.2, J70.2-J70.4, K85.3, L10.5, L27.0, L27.1, M10.2, M32.0, M80.4, M81.4, M83.5, M87.1, R50.2
Mental/behavioral disorders due to drugs	(F11.0-F11.5, F11.7-F11.9, F12.0-F12.5, F12.7-F12.9, F13.0-F13.5, F13 .7-F13.9, F14.0-F14.5, F14.7-F14.9, F15.0-F15.5, F15.7-F15.9, F16.0-F16.5, F16.7 -F16.9, F18.0-F18.5, F18.7- F18.9, F19.0-F19.5, F19.7-F19.9
Alcohol-induced illnesses	E24.4, G31.2, G62.1, G72.1, I42.6, K29.2, K70.0-K70.4, K70.9, K73.4, K85.2, K86.0, R78.0
Mental/behavioral disorders due to alcohol	F10.0-F10.9
Accidental or intentional poisoning and poisoning of undetermined intent from alcohol exposure	X40-45, X65, Y10-15, Y45, Y47, Y49
Suicide	X66-X84, Y87.0

### Independent variables

We consider as independent variables sex (male and female); age (divided into decennial groups in adulthood, from the ‘20s to the last group of over 80); and race, divided into whites, blacks, and browns. Our study was particularly interested in racial issues involving blacks and browns. In addition, the occurrence of indigenous and Asian people is shallow [22]. For this reason, we chose to exclude these racial categories.

First, Case and Deaton’s original study [14] adequately describes the American population and deaths of despair by sex, age, race, and education. The absence of education information in almost half of the death records in Brazil limits the analysis capacity of the present study compared with the original North American study. However, it is essential to consider that, unlike in the United States, the number of Brazilians with higher education is only 16.5% [23]. This low frequency would potentially create a floor effect, reducing intergroup variability due to the high concentration of people in the low education categories. This phenomenon may compromise the property of this indicator to discriminate the populations under comparison. Thus, even if this information were available, it might not be the best criterion for analyzing the Brazilian people.

### Adjust mortality levels and pattern

As it is a very heterogeneous set of municipalities, to ensure data quality, we adjusted mortality estimates for underreporting, age errors, and ill-defined causes [24,25]. We adjusted for the effect of underreporting on mortality rates by applying indirect estimation methods to the 2000 and 2010 census data. The techniques used were Synthetic Extinct Generation (SEG) and General Growth Balance (General Growth Balance, GGB) in combination [26] to estimate adult mortality.

Secondly, we corrected the mortality data using the methods described for the Mortality Information System (SIM) set of deaths. We considered only deaths from the above causes based on the duly updated data. We corrected deaths of despair records by proportional redistribution by year and age group of deaths classified as ill-defined causes, using the methodology proposed by the World Health Organization (WHO) [27]. For more information concerning adjustment techniques application, see Supplementary File #1).

### Data analysis

First, we explored the time series through statistical tests that could confirm a trend to analyze there [28]. We apply the Wald-Wolfowitz test to verify the stationarity hypothesis of the historical series. We compare the median value of the historical series with each point in time, forming pairs of comparisons. The number of positive pairs (when the point estimate is greater than the median) and opposing pairs (when the point estimate is less than the median) determines whether there are variations in time. The test statistic evaluates the number of pairs where the point values differ from the median. Thus, we reject the null hypothesis (H_0_) when a few pairs are similar to the median.

Then, we performed the Cox-Stuart test to obtain the trend effect. Based on the binomial distribution, this method groups the observations in pairs. Each pair (X_i_, X_i + c_) associates the "+" sign if X_i_ < X_i + c_, and the "-" sign if X_i_ > X_i + c_, eliminating ties, for c = N/2, where N is the number of observations in the series, and Xi is the observation (i = 1,. . ., N).

The test verifies the following hypothesis:

$$H_{0}:P(X_{i}<X_{i+c})=P(X_{i}<X_{i+c})\;\forall i;\;H_{1}:P(X_{i}<X_{i+c})\neq P(X_{i}<X_{i+c})\;\forall i$$

The hypotheses test the existence of a trend (H_1_) or not (H_0_). There is no trend if the probability of "+" signs equals the likelihood of "-" signs.

After this first diagnosis, we performed the interrupted time series analysis [29]. The first decision when considering an interrupted time series analysis is whether it is an appropriate design for the specific assessment in question. We assumed this was a natural experiment whose intervention was not controlled. We could differentiate between two periods: before and after the economic stagnation (2011). We assumed that there is a level change in the trend occurring without delay, that is, without temporal lag. It suggests that the effects of the economic crisis and austerity measures are taking place quickly.

For the specific mortality rate by cause group, we first analyzed time trends for the years before and after economic stagnation (2003 to 2011) and the worsening of the crisis (2011 to 2018). To avoid violating the principles of homoscedasticity and the independence of residuals in the time series analysis, we chose not to use linear regression but the Prais-Winsten method [30]. It is a model that belongs to the class of generalizable linear models whose serial autocorrelation is defined from the dependence of a serial measure, evaluated by the Durbin-Watson statistics [31].

After, we compared the slope of the linear trend. We tested the null hypothesis that there was a break in the direction of the time series in the year 2011. For this, we used a trend break regression model, which is common in the literature, to analyze structural breaks [32]. The model tested was:

$$H_{t}=\alpha +\beta_{2}*t+\beta_{2}*(t-2011)*D+\varepsilon_{t}$$

Where H_t_ is a health indicator for year t, D is a dummy variable where D = 0 if t <2011 and D = 1 if t> 2011. The term (t—2011) *D is a linear spline specification that breaks the time trend in 2011. The β_2_ coefficient allows a direct statistical test of the slope change of Ht. The p-value corresponds to the test in which the null hypothesis is β_2_ = 0 (therefore, no change in trend).

As is often the case in population health assessments, here, the result is a count, and we used a Poisson regression model without loss of generality. In addition, the age-standardized population was included as a compensation variable to transform the result into a rate and adjust for possible changes in the population over time. To avoid overdispersion, we used robust variance to estimate the standard error. Finally, we evaluated the autocorrelation through residual analysis and the partial autocorrelation function.

### Ethics approval

The study used secondary databases. The information is public and does not contain sensitive data that allows people to be identified. Following national (Resolution 466/2012 and 510/2016) and international (Declaration of Helsinki) ethical legislation on research involving human beings, an ethics committee exempts the present study from consideration.

## Results

**Fig 1:**
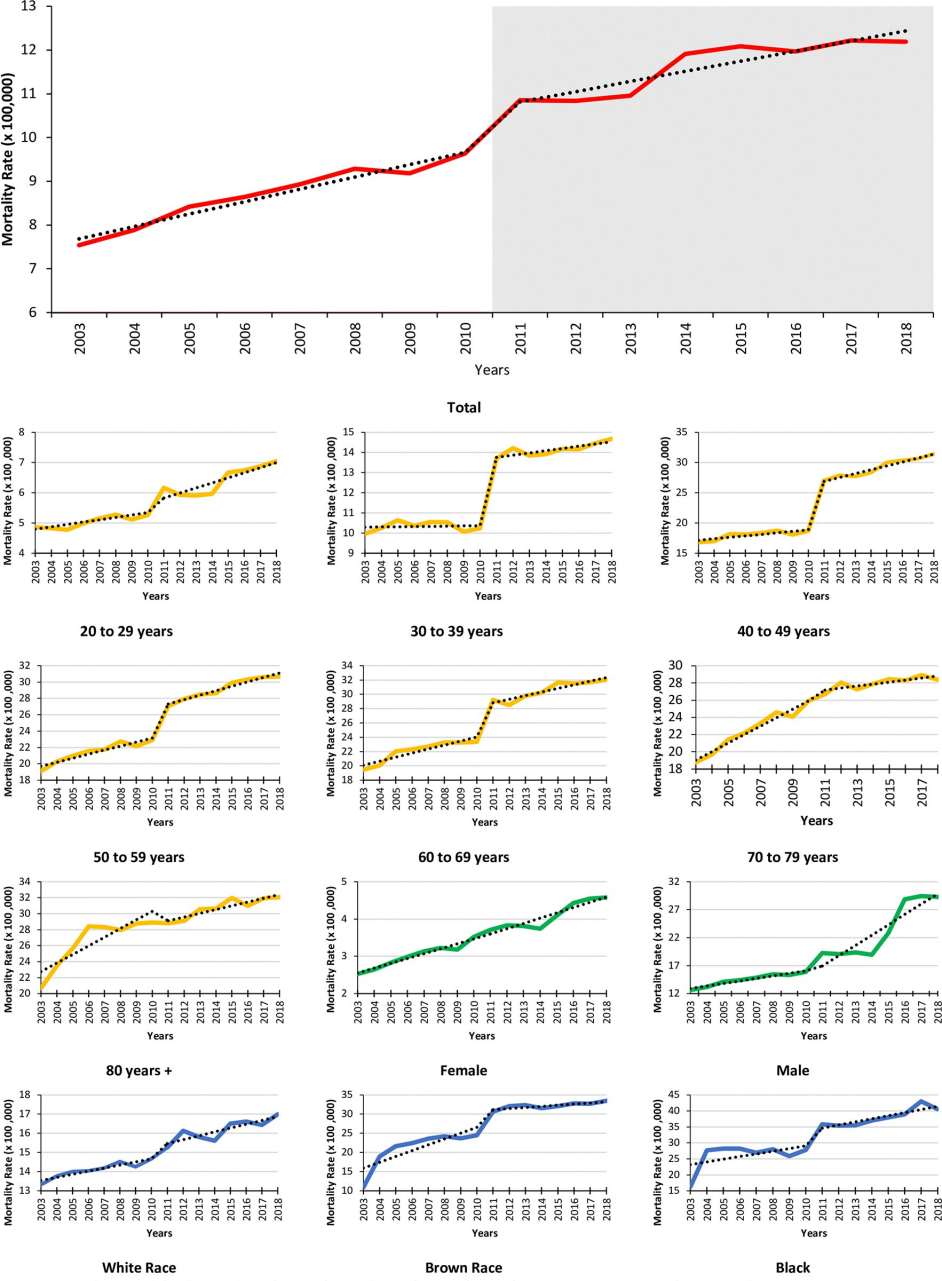
Historical series of death rates from despair during the study period according to sex, age group, and race. Brazil, 2003 to 2018. **Source:** Mortality Information System (SIM/SUS) | National Bureau of Statistics (IBGE).

Visual inspection of the historical series of deaths of despair rates suggests a progressive increase over the years. ure 1 provides some graphical evidence that the rise in the mortality rate was unstable over the entire study period. There was an immediate increase in level from 2011 onwards, with a slight deceleration of the upward trend. Due to the lag period between the onset of the recession and changes in mortality trends, a visual inspection of the data suggests that a gradual and continuous effect may be observed in all categories of explanatory variables. In addition, for some of them, it is possible to observe an immediate change in the level of the historical series (Fig 1).

We diagnosed randomness using the Wald-Wolfowitz test (Table 2). The analysis of the series parameters (mean and variance) and the verification of statistical significance (α = 0.05) rejected the null hypothesis of the randomness of the data. In this way, it becomes possible to attempt to model the data. Then, to verify the effect of the trend, we performed the Cox-Stuart test. The analyzed series show an increasing tendency, with a significant test (i.e., we reject the null hypothesis of a stationary series at a significance level of 5%). Preliminarily, the upward trend is linear for all variables.

**Table 2:** Diagnosis of randomness and trend effect of historical series of death rates due to despair according to explanatory variables. Brazil, 2003–2018.

Categories	Randomness (Wald-Wolfowitz Test)								Trend Effect (Cox-Stuart test)						
	R	n_0_	n_1_	n	E(R)	Var(R)	DP(R)	p-value*	β_1_	EP_res_	gl	R^2^	F	Trend	p-value**
**Age Group**															
20 to 29 years	2	8	8	16	9	3.733	1.932	<0.001	0.159	0.235	14	0.911	155.3	increasing	0.004
30 to 39 years	2	8	8	16	9	3.733	1.932	<0.001	1.001	0.918	14	0.801	56.52	increasing	0.004
40 to 49 years	2	8	8	16	9	3.733	1.932	<0.001	1.160	2.112	14	0.880	1030	increasing	0.004
50 to 59 years	2	8	8	16	9	3.733	1.932	<0.001	0.859	1.035	14	0.944	234.4	increasing	0.004
60 to 69 years	2	8	8	16	9	3.733	1.932	<0.001	0.932	1.254	14	0.931	188.1	increasing	0.004
70 to 79 years	2	8	8	16	9	3.733	1.932	<0.001	0.669	1.035	14	0.910	141.9	increasing	0.004
80 years +	2	7	9	16	8.875	3.609	1.900	<0.001	0.590	1.419	14	0.808	58.54	increasing	0.004
**Sex**															
Female	2	6	10	16	8.5	3.250	1.803	<0.001	1.359	0.111	14	0.973	506.2	increasing	0.004
Male	2	8	8	16	9	3.733	1.932	<0.001	1.120	2.277	14	0.857	82.26	increasing	0.004
**Race**															
White	2	8	8	16	9	3.733	1.932	<0.001	0.239	0.294	14	0.941	224.9	increasing	0.004
Brown	2	8	8	16	9	3.733	1.932	<0.001	0.657	1.030	14	0.908	138.5	increasing	0.004
Black	2	8	8	16	9	3.733	1.932	<0.001	0.662	1.477	14	0.830	68.47	increasing	0.004
**Total**	2	8	8	16	9	3.733	1.932	<0.001	0.341	0.321	14	0.962	382.6	increase	0.004

R–number of observation sets; n_0_ –number of observations below the period average; n_1_—number of observations above the period average; n–total number of observations of the distribution; E(R)–expectation of the number of groups of observations; Var(R)–variance of the number of observation groups; SD(R)–standard deviation of the number of observation groups; Z–Wald’s test for statistical significance. β_1_ –coefficient; SE_res_−standard error of the model residue; R^2^ –coefficient of determination, F–model test statistic

* p-value for Wald Wolfowitz test

** p-value for Cox-Stuart test.

**Source:** Mortality Information System (SIM/SUS) | National Bureau of Statistics (IBGE).

The results in Table 2 reveal significant upward trends in deaths of despair across various sociodemographic categories in Brazil from 2003 to 2018. Age groups, ranging from 20–29 to 80+, exhibit statistically significant increasing trends, with notable intensification in middle-aged groups (30–59 years) compared to older adults, indicating a substantial impact of economic cycles on younger populations. Sex-wise, both males and females show significant upward trends, but males exhibit a higher trend coefficient, aligning with findings that men are particularly vulnerable to economic shifts. Racial analysis indicates an increase in deaths for all groups, with Black and Brown populations experiencing notable upward trends, suggesting disproportionate effects of economic adversity on marginalized communities. These findings underscore the selective impact of economic crises on different demographic segments, providing crucial insights into targeted intervention needs.

Table 3 highlights significant differences in deaths of despair mortality rates in Brazil across sociodemographic variables before and after economic stagnation (2003–2011 vs. 2011–2018), supporting the study’s focus on the impact of economic cycles.

**Table 3:** Descriptive statistics on mortality rates by sex, age group, and race. Brazil, 2003–2018.

Variables	Stage of the economic cycle				p-value^a^
	Before economic stagnation (2003 to 2011)		After the economic stagnation (2011 to 2018)		
	Average	SD	Average	SD	
**Age Group**					
20 to 29 years	5.034	0.197	6.411	0.464	<0.001
30 to 39 years	10.328	0.240	14.146	0.321	<0.001
40 to 49 years	17.999	0.719	29.178	1.618	<0.001
50 to 59 years	21.428	1.281	29.203	1.356	<0.001
60 to 69 years	22.063	1.505	30.570	1.324	<0.001
70 to 79 years	22.490	2.460	30.000	0.707	<0.001
80 years +	26.524	3.014	30.760	1.265	0.003
**Sex**					
Female	3.005	0.325	4.093	0.374	<0.001
Male	14.481	1.131	23.375	4.984	<0.001
**Race**					
White	14.099	0.424	16.170	0.576	<0.001
Brown	13.294	1.436	19.326	0.517	<0.001
Black	16.483	0.493	22.805	1.616	<0.001
**Total**	8.689	0.717	11.628	0.626	<0.001

SD–standard deviation

^a^ p-value obtained from Student t-test (for dichotomous variables) and ANOVA test for variables with three or more categories.

**Source:** Mortality Information System (SIM/SUS) | National Bureau of Statistics (IBGE).

For age groups, mortality rates sharply increased with age. They were notably higher in all age categories post-stagnation, with the 40–49, 50–59, and 60–69 groups showing exceptionally high average rates after 2011 (e.g., the 40–49 group rose from 17.999 to 29.178). This trend suggests increased vulnerability among middle-aged and older adults during economic downturns.

On the Other hand, sex differences show a substantial rise in male mortality rates (from 14.481 to 23.375). At the same time, females exhibited lower but still statistically significant increases (from 3.005 to 4.093), indicating that men were disproportionately affected.

In terms of race, the Brown and Black populations experienced the most marked increases, with Black individuals’ rates rising from 16.483 to 22.805 and Brown individuals’ rates increasing from 13.294 to 19.326. The White population saw a smaller but significant increase from 14.099 to 16.170. It indicates that socioeconomic and racial disparities may exacerbate the effects of economic adversity, aligning with the study’s objectives to explore how economic crises affect deaths of despair across diverse demographics.

Table 4 details the trend in deaths of despair rates across sociodemographic variables in Brazil before and after economic stagnation (2003–2011 vs. 2011–2018), showing how the economic cycle affected these rates. Increases in trend coefficients (β) were observed across all age groups post-stagnation, with the 40–49 and 50–59 age groups showing significant increases in mortality rates, reflecting heightened vulnerability in middle-aged adults. The coefficient of determination (R^2^) values for these groups remained high, indicating robust model fit, with some reaching over 0.97, suggesting pronounced mortality rate trends across the phases of the economic cycle. Likewise, male mortality trends displayed a substantial increase in β (from 0.452 to 1.714), indicating a more significant impact of economic stagnation on men than women. The change in trend for women was smaller but significant, with β shifting from 0.128 to 0.136 post-stagnation.

**Table 4:** Trend in deaths of despair rates before and after the onset of economic stagnation. Brazil, 2003–2018.

Variables	Before economic stagnation (2003 to 2011)						After economic stagnation (2011 to 2018)					
	β	SE	p-value	R^2^	F Statistic	DW	β	SE	p-value	R^2^	F Statistic	DW
**Age Group**												
20 to 29 years	0.070	0.016	<0.001	0.732	16.37	1.867	0.161	0.040	0.007	0.781	21.34	1.604
30 to 39 years	0.014	0.043	0.009	0.702	14.12	1.655	0.106	0.025	0.006	0.984	363.20	1.931
40 to 49 years	0.249	0.065	0.027	0.585	80.46	1.890	0.645	0.057	0.000	0.969	187.70	2.059
50 to 59 years	0.493	0.069	<0.001	0.914	63.94	1.652	0.542	0.046	0.000	0.986	429.30	1.663
60 to 69 years	0.561	0.124	0.001	0.881	44.51	1.483	0.516	0.071	0.000	0.976	241.50	1.824
70 to 79 years	0.981	0.070	0.002	0.985	391.10	2.000	0.233	0.029	0.000	0.999	1186.00	2.245
80 years +	1.138	0.324	0.024	0.602	90.09	1.495	0.472	0.074	0.001	0.979	281.60	1.821
**Sex**												
Female	0.128	0.009	<0.001	0.989	528.60	2.078	0.136	0.028	0.003	0.878	43.25	1.393
Male	0.452	0.047	0.001	0.871	40.65	1.742	1.714	0.425	0.007	0.677	12.59	1.427
**Race**												
White	0.155	0.020	<0.001	0.995	1.20	1.791	0.189	0.036	0.002	0.993	833.60	2.484
Brown	0.543	0.110	0.004	0.769	19.92	1.539	0.170	0.038	0.004	0.985	392.20	1.994
Black	0.071	0.005	<0.001	0.990	608.70	1.806	0.624	0.098	0.001	0.969	184.60	1.885
**Total**	0.287	0.027	<0.001	0.915	64.81	1.824	0.227	0.048	0.003	0.779	21.14	1.745

β–linear coefficient; SE–standard error; R2 –coefficient of determination; DW–Durbin Watson Test.

**Source:** Mortality Information System (SIM/SUS) | National Bureau of Statistics (IBGE).

Furthermore, the black population saw the most significant post-stagnation increase in β (from 0.071 to 0.624), suggesting that economic downturns disproportionately impacted Black individuals. Brown and White populations also showed increased trends but to a lesser degree. The R^2^ values remained high, especially for White and Black populations, indicating consistent trend reliability.

Assuming these are robust-modeling series, this difference is due to a change in the level or pattern of the trend, suggesting a discontinuity in the trend for some classes. We then used the interrupted time series technique to verify the change hypothesis (Table 5).

**Table 5:** Effect of economic stagnation on the time series of deaths of despair rates. Brazil, 2003–2018.

Variables	Categories	β	SE	p-value	R^2^	F Statistic	DW
**Age group**	**20 to 29 years**						
	Level	0.917	0.247	0.003	0.9762	164.10	1.780
	Trend	-0.061	0.056	0.300			
	**30 to 39 years**						
	Level	0.450	0.198	0.042	0.9366	59.11	1.841
	Trend	0.097	0.046	0.055			
	**40 to 49 years**						
	Level	3.281	0.232	<0.001	0.9885	344.40	1.765
	Trend	0.099	0.051	0.007			
	**50 to 59 years**						
	Level	7.408	0.401	<0.001	0.9965	1.13	1.967
	Trend	0.397	0.086	0.001			
	**60 to 69 years**						
	Level	3.617	0.398	<0.001	0.9931	575.20	1.711
	Trend	0.047	0.086	0.596			
	**70 to 79 years**						
	Level	4.359	0.617	<0.001	0.9837	242.00	1.879
	Trend	-0.068	0.137	0.627			
	**80 years +**						
	Level	1.098	0.357	0.010	0.9962	1035.00	1.886
	Trend	-0.730	0.072	<0.001			
**Sex**	**Female**						
	Level	0.015	0.130	0.910	0.9453	69.07	1.637
	Trend	0.009	0.032	0.783			
	**Male**						
	Level	0.424	1.793	0.817	0.859	24.36	1.486
	Trend	1.307	0.479	0.018			
**Race**	**White**						
	Level	0.698	0.193	0.004	0.994	657.20	2.356
	Trend	0.042	0.039	0.307			
	**Brown**						
	Level	3.348	0.483	<0.001	0.9635	105.60	1.623
	Trend	-0.369	0.124	0.011			
	**Black**						
	Level	3.697	0.563	<0.001	0.9862	285.30	2.079
	Trend	0.654	0.115	<0.001			
**Total**	Level	0.917	0.247	0.003	0.9762	164.10	1.780
	Trend	-0.061	0.056	0.300			

β–linear coefficient; SE–standard error; R2 –coefficient of determination; DW–Durbin Watson Test.

**Source:** Mortality Information System (SIM/SUS) | National Bureau of Statistics (IBGE).

The beginning of economic stagnation caused immediate changes for all age groups and all races. Still, it did not significantly cause this effect for any sex, at a level of 95% (p-value = 0.910 for males and 0.817 for females). This observation is related to the coefficients of the variable "level" coefficient, highlighted in Table 5. Regarding the gradual change over time (coefficient of the variable "trend"), this effect was observed for people aged 40 to 49 years (p-value = 0.007), 50 to 59 years (p-value = 0.001), 80 years and over (p-value <0.001), men (p-value = 0.018), browns (p-value = 0.011) and blacks (p-value <0.001). With this, it is possible to say that population subgroups suffered an immediate effect of the crisis, but the trend adjusted over time after the beginning of the crisis. It is what seems to occur for the entire population as a whole. However, we have two other scenarios: groups for which there was no immediate change, but the deepening of the crisis has gradually impacted mortality rates (non-significant level and significant trend), as it happens for men (p-value level = 0.817; p-value trend = 0.018); and groups in which the effect was immediate, and has been increasing with increasing depth (significant level and trend), as is the case of blacks (p-value level<0.001; p-value trend<0.001) and browns (p-value level<0.001; level<0.001; p-value trend = 0.011). Ultimately, these results suggest that recovery is selective for specific groups.

## Discussion

The historical series of deaths of despair shows an increasing trend, with a significant rise after 2011 linked to economic stagnation. Statistical tests confirmed non-random upward trends, with notable differences before and after 2011. An interrupted time series analysis highlighted immediate mortality rate increases for most demographic groups post-2011, except by sex, where changes were gradual. Subgroups such as specific age groups and Black and Brown populations exhibited both immediate and progressive effects, while men showed delayed impacts. These findings suggest that economic downturns selectively impacted mortality rates, making specific demographics more vulnerable.

In fact, over the last few years, Brazil recorded an increase in suicide rates, especially following the global economic downturn (2008–2013) and political crisis (2014–2017), with some subgroups affected more acutely over time. In general, males (78.84%) and people aged 30–44 had the highest suicide rates. Trends by race vary according to region. It’s important to mention that race distribution by regions is also unequal, partially explaining differences between national and subnational trends [33,34].

The narrative surrounding the distribution of wealth can serve as a lens through which to understand a nation’s broader historical trajectory [35]. Similarly, examining the dysfunctional mechanisms that exacerbate disparities aids in elucidating distinctions between nations. Despite debates regarding their applicability, insights into deaths attributed to despair have spurred scientists, politicians, and the populace to acknowledge the impact of economic circumstances on health outcomes. Brazil is no exception to this paradigm. Thus, it becomes imperative to delineate the historical ebbs and flows of economic cycles within the country and grasp their correlation with deaths of despair fatalities.

Since mid-2014, Brazil’s economic status officially transitioned into a recessionary phase. This downturn stemmed from economic policies initiated in 2011. These policies contributed to a decline in productivity, triggering a domestic debt crisis, followed by measures to curb inflation that tightened monetary policy. This comprehensive approach involved reducing interest rates, implementing fiscal policies geared toward investment, augmenting expenditure, offering subsidies, and intervening in pricing structures [36].

Consequently, the strategy led to a diminishment in the nation’s economic growth potential. This scenario was compounded by certain factors, such as a surge in informal employment accompanied by layoffs. Despite a recent uptick in GDP, it failed to instill confidence among entrepreneurs and investors [37]. Moreover, there was a halt in net capital accumulation within the Brazilian economy. The drive to reduce business overheads to foster market competition solidified the recessionary state, exacerbated from 2015 onwards. Termed fiscal adjustment, this policy package aimed to curtail government spending through tax administration reforms, austerity measures, and structural overhauls [38]. Consequently, the recession manifested in a deterioration of public indebtedness.

Brazil’s ongoing fiscal austerity measures since 2016 pose economic and social hazards to the populace. Apart from the adverse impacts on overarching economic indicators, an austerity plan was enacted to freeze social expenditure for two decades. Consequently, this move led to a 3.1% contraction in healthcare and education outlays. These reforms jeopardize establishing a robust Brazilian welfare system [19,39]. Notably, the disadvantaged segments of the population bear the brunt of this adverse environment. It perhaps explains the stark rise in deaths attributed to despair, particularly among the black and brown communities, males, and middle-aged adults. Furthermore, Brazil’s economic crisis and fiscal austerity measures may engender a more dire scenario than developed nations. It’s worth noting the country’s persistently high levels of social inequality, underinvestment in the healthcare sector, elevated prevalence of chronic diseases, and the persistence of preventable infectious ailments.

The response strategies hinge upon the ideological leanings of governing bodies [4]. In this context, the conceptualization of the austerity model stems from policymakers’ perception of state size, directly impacting public health as it may not necessarily bolster the welfare state’s growth. However, avenues for addressing these challenges are limited. It’s crucial to contextualize that Brazil has been embroiled in a political crisis since 2014, concurrently with the economic turmoil. This crisis intensified with the impeachment proceedings against President Dilma Rousseff in 2016. Even after her removal, the political landscape remained fraught, exacerbated further by policies implemented by the Bolsonaro administration since 2018, further eroding the welfare state [40].

The global trend in deaths of despair mortality rates indicates a correlation with economic fluctuations. While the theoretical underpinning of deaths of despair is intertwined with psychopathological models, its relevance lies more prominently within the framework of social determinants of health [41]. This linkage primarily stems from attempts to associate it with contextual factors, particularly socioeconomic ones. Hence, underscoring the relationship with covariates, especially race, is imperative. Literature addressing deaths of despair from a social determinants perspective remains relatively sparse, with specific determinants still underexplored.

Nonetheless, existing evidence suggests significant disparities in how deaths of despair impact various subpopulations, particularly African American and Hispanic communities [42]. The seminal study by Case and Deaton [14] noted a more pronounced upward trend among the white population. A study conducted in Canada revealed a similar pattern, albeit with migration status emerging as a crucial confounding factor, suggesting that within migrant populations, the white race didn’t pose a heightened risk [43]. It’s also noteworthy that the National Center of Health Statistics uncovered disparities in reporting, with overestimations in white and black populations (1% and 5%, respectively), potentially linked to historical undercounting of specific demographics, including black and Hispanic populations in mortality rate calculations [44].

In our analysis, we observed a more pronounced impact of the economic crisis on deaths of despair among individuals of African descent (black and brown race). Brazilian society’s foundation rests on the historical enslavement of African peoples. The abolition of slavery, devoid of any socioeconomic reparations, relegated the previously enslaved black population to a state of sub-citizenship characterized by dismal health, employment, and educational metrics compared to the white population [45,46], rendering them more susceptible to the repercussions of economic and health crises. Despite the compelling narrative surrounding the psychosocial ramifications of downward mobility among middle-aged white working-class individuals, the notion that it poses a risk solely to this demographic segment reflects a biased worldview within specific segments of the US population [47]. Such assumptions can obscure the focus on the effects of discriminatory political and economic practices, segregation, and oppression experienced by racialized and ethnic working-class minorities [48].

Our study has certain limitations. The two periods encompass dynamic changes. The period from 2003 to 2011 coincided with the acute phase of the global crisis. However, preliminary analyses indicate that its impact on Brazil was not significant until 2011. Furthermore, the second analysis period (2011 to 2018) encapsulates deteriorating economic indicators and federal interventions through fiscal austerity measures. While this period could be subdivided, the limited number of observations renders interrupted time series analyses unfeasible.

Still concerning the time series, we decided to analyze 2003–2018, although mortality data is available up to 2022. This decision is based on choosing the same time range before and after the period effect. 2003 was a political mark in Brazil due to the change of government into a left-wing phase. Aligning with that, if we considered including 2019 in the time range, we should include 2002 as well, and we believe it could confound the analysis overall. It was also a choice not to consider the time during the COVID-19 pandemic (and the equivalent amount of time before the stagnation beginning in 2011) since it interferes with many health outcomes such as suicides, which is the majority of deaths of despair.

Due to a lack of quality data, we did not analyze disaggregating mortality data by educational level, as Case and Deaton [14] did. However, it’s important to mention that the distribution of educational levels in Brazil is much different in Brazil than in the US. Recent data from IBGE shows that 53% of the entire population has only primary education, so most likely, the education level would have a poor discrimination power in this analysis. Finally, concerning population, the 2010 and 2022 Census showed a growth in the population, particularly the Black population. It might impact the magnitude of mortality rates.

## Conclusion

The objectives of this study were to assess the impact of Brazil’s economic crisis and austerity measures on deaths of despair and to analyze their trends from 2003 to 2018, considering different phases of the economic cycle. Our findings reveal a consistent increase in deaths of despair, especially after 2011, correlating with economic stagnation. Interrupted time series analysis identified significant changes in mortality rates across demographic groups, with immediate effects in specific populations, notably Black and Brown communities, middle-aged adults, and men, highlighting the selective vulnerability of particular demographics.

Our study contributes to understanding socioeconomic determinants of health in Brazil, demonstrating the profound impact of economic cycles on mental health and mortality rates. By linking economic crises and austerity policies to increased deaths of despair, particularly among vulnerable groups, these findings underscore the need for comprehensive public health policies that mitigate the effects of economic instability. Our analysis suggests that addressing economic disparities and implementing supportive interventions for high-risk populations could reduce deaths of despair, informing future policy responses during economic downturns. Hence, it’s prudent to highlight Brazilian findings aligning with Case and Deaton’s diagnosis.

## Supporting information

S1 Text Correction of mortality levels. (PDF)
