# Supplementary material for: The effect of economic crisis and austerity measures on deaths of despair in Brazil: An interrupted time series analysis
Source: PLoS One. 2024 Dec 31;19(12):e0314294. doi: 10.1371/journal.pone.0314294 (PMC11687905; doi:10.1371/journal.pone.0314294)
Supplement: S1 Text — (PDF) [file pone.0314294.s001.pdf]

SUPPORTING INFORMATION FILE #1

Correction of mortality levels

Initial settings

Studying the level and pattern of mortality and obtaining reliable estimates is essential to understand demographic dynamics. In developing countries, data quality has always compromised pattern estimation and knowledge of mortality levels. In the case of deaths, there are two main problems: the underreporting of deaths and the misclassification of causes of death. Both compromise the consistency and proper use of the registration of deaths from other causes. In this sense, it is always important to diagnose this quality to assess the need for correction when underreporting or non-random errors exist in the distribution of births and deaths, for example, by age group and sex. Therefore, the low quality of vital records in some regions makes it difficult to estimate mortality, and the analysis of the profile of causes of death is complicated. Therefore it is necessary to correct the ends.

In Brazil, analyzing the quality of information available in official statistics often becomes challenging. There is misreported information from household surveys and administrative records, such as mortality data obtained by the Mortality Information System (SIM). For all of them, there is a recognized improvement in the quality of known information and its coverage[1]. However, this improvement is not uniform across countries or regions.

Correction requires two steps: correction of the level of mortality (predicting an under-enumeration of total deaths)[2], and correction of information on causes of death, due to failure to fill in the cause of death, in the case of death reported as an ill-defined cause, or filling out garbage codes[3,4].

For the present analysis, we used death data from Brazil for 2000 and 2010, obtained by the Mortality Information System (SIM/SUS). Additionally, we got data referring to the population by age group from census data from the IBGE for the same period. In general, by comparing the distribution of deaths by age and the age distribution of the population, it is possible to obtain the age pattern of mortality in a time interval, including the displacement of this pattern along the cohorts, in typically not stable populations[5].

Level correction for deaths

Among the techniques used to assess the death record, three stand out: the balancing equation, General Growth Balance (GGB)[6]; that of extinct generations, Synthetic Extinct Generation (SEG) (Bennet & Horiuchi, 1981); and the Adjusted Extinct Generations

---

[1]MELLO JORGE MHP, LAURENTI R, GOTLIEB SLD. Análise da qualidade das estatísticas vitais brasileiras: a experiência da implantação do SIM e do SINASC. Rev Ciência Saúde Coletiva 2007, 12(3): 643-54.

[2]QUEIROZ BL, FREIRE FHMD, GONZAGA MR, LIMA EEC. Estimativas do grau de cobertura e da mortalidade adulta (45q15) para as unidades da federação no Brasil entre 1980 e 2010. Rev. Bras. epidemiol. 2017; 20(suppl.1): 21-33.

[3]MATHERS CD, FAT DM, INOUE M, RAO C, LOPEZ AD. Counting the dead and what they died from: an assessment of the global status of cause of death data. Bull World Health Organ. 2005; 83(3): 171-177.

[4] LAURENTI R, MELLO-JORGE MHP, GOTLIEB SLD. Mortalidade segundos causas: considerações sobre a fidedignidade dos dados. Rev Panam Salud Publica. 2008; 23(5):349-356.

[5]MOULTRIE T, DORRINGTON R, HILL A,HILL, K, TIMAEUS I, ZABA B. Tools for Demographic Estimation. Paris: International Union for the Scientific Study of Population, 2013. Available at http://demographicestimation.iussp.org. Accessed in November 2021.

[6] HILL, K, YOU D, CHOI Y. Death distribution methods for estimating adult mortality: sensitivity analysis with simulated data errors. Demographic Research. 2009; 21(9): 235-254.

(SEG-adj)[7]. In all of them, some presuppositions cannot be ignored: it is considered, first, that the population is closed; in addition, it is assumed that the degree of coverage of deaths is constant by age; still, the degree of coverage of the population count is consistent by age; finally, the ages of the living and the dead are declared without errors. It is worth noting that these methods use data from two different periods to capture changes that occurred over time, indirectly estimating the cohort effect, not just the period effect. In other words, the advantage of these three methods concerning the previous formulations for correcting the underreporting of deaths is precisely the flexibility of the assumption of a stable population.

General Growth Balance Method (GGB)

The GGB method is based on the compensating equation; that is, it defines the growth rate from the difference between the entry and exit rates of the population. Since this is a closed population, entries are represented by births and exits by deaths. In this case, if the mortality estimate can be estimated from two population censuses and compared with a direct mortality estimate using the registration or enumeration of deaths from the demographic census, the degree of coverage of the death registry can be estimated from the relationship between these two quantities. Therefore, it is possible to estimate the variation of coverage for the analyzed period.

The first step of the method consists of accumulating the population (N(x+)) in the two periods and the deaths (D(x+)) from the most advanced age groups to the youngest in each age group.

It is considered that A is the initial age of the open interval, and x is the initial age of each interval, varying every five years, starting from zero. The calculation is done similarly for deaths. Next, it is necessary to calculate an estimate of person-years (L(x)) in each age group. The time measures considered for this calculation are t1 and t2, representing the initial and final observation period (in this case, 2000 and 2010). With this information, it is possible to estimate the number of people born in the period. The relationship between the difference between the inflows and the growth rate (e(a+) – r(a+)) and deaths (d(a+)) is expected to be perfect. Any discrepancy between these indicators represents coverage problems. It is possible to estimate an intercept that captures any variation in coverage between the two censuses from the relationship between the difference in the entry rate and the growth rate with the mortality rate in each age group. Also, we can estimate a slope that indicates the degree of coverage of the death record concerning the average coverage of both censuses[8].

Figure 1 explains this relationship in Brazil between 2000 and 2010. For better performance of the method, ages between 5 and 65 years are considered for the estimates of the correction factor of mortality rates due to random fluctuations in the number of deaths or the population in some age groups. After that, we excluded the points and then calculated the standard deviation to calculate the degree of data coverage between the years 2000 and 2010. We highlight the two outliers are represented by the extreme age groups, with an essential under-enumeration in the 0 to 4 years range and an equally important over-

[7] BENNET NG, HORIUCHI S. Mortality Estimation from Registered Deaths in Less Developed Countries. Demography. 1984; 21(2): 217-233.

[8] QUEIROZ BL, SAWYER DO. O que os dados de mortalidade do Censo de 2010 podem nos dizer? Revista Brasileira de Estudos de População. 2012; 29(2): 225-238.

numbering in the open range of 80 years and over (dark highlighted points). We exclude these groups from the calculations.

Regarding adult mortality, for males, there is a slight under-enumeration in the groups between 20 to 24 and 30 to 34 years old, with points below the expected line. The age groups between 35 and 39 years old to 70 to 74 years old remained very close to the line adjusted by the selected points, and the group from 79 to 75 years old shows a slight over-enumeration. It is important to emphasize, however, that these differences do not justify the exclusion of these bands. For women, a similar sub-enumeration relationship can be observed for the groups between 30 and 34 years old and 40 to 44 years old. There was no overestimation for any age group except for the one previously excluded. We emphasize that the information is of good quality, especially for 2010.

It is important to note, therefore, that there were no outliers in the adult age group. The line that describes the expected number of deaths is stable at almost all age group points. This is possible since we use data from the Mortality Information System, not Population Censuses. For these data, since the 2000s, there has been a federative pact (therefore, involving the federal government, states, and municipalities to reduce the magnitude of ill-defined causes and the active search for death records[9,10], with overall coverage estimated at more than 95% between 2000 and 2010, and with a reduction of more than 50% in ill-defined causes[11]. Intercensal coverage was 99.1% and 98.9%, respectively, for men and women.

Synthetic Extinct Generations (SEG) Method

Bennett and Horiuchi's (1981) method, called the extinct generations (SEG) method, uses age-specific growth rates to convert a population's age distribution of deaths into an age distribution. The author argues that, in a stationary population, the deaths observed from age x are equal to the population at age x, adjusted by the population growth rate by age range. In this way, the deaths of a population at age x+ estimate the population at age x. The age-specific population growth rates are then used to adjust the number of deaths from the stationary population to a non-stable population. Finally, the degree of coverage of the death registry corresponds to the ratio between the estimated deaths by the population over age x and the population observed over age x.

There are differences in the adjustment of each track. First, growth within the age group is calculated. Second, the effect of net migration is subtracted, while a term is added that represents the annual growth rate between 2000 and 2010. The estimate is made from interpolation using model tables for the open interval. We then calculated the degree of coverage of deaths. First, we computed coverage within each age group. We calculated the total coverage from each initial age interval to the open interval. Finally, we defined the degree of coverage, specifying the age interval used for this purpose. For Brazil, we considered the range from 20 to 74 years.

---

[9] ALMEIDA WS, SZWARCWALD CL. Adequação das informações de mortalidade e correção dos óbitos informados a partir da Pesquisa de Busca Ativa. Ciência & Saúde Coletiva. 2017; 22(10): 3193-3203.

[10] FRIAS PG, SZWARCWALD CL, LIRA PIC. Avaliação dos sistemas de informações sobre nascidos vivos e óbitos no Brasil na década de 2000. Cad Saude Publica. 2014; 30(10):2068-2080.

[11] LIMA EE, QUEIROZ BL. Evolution of the deaths registry system in Brazil: associations with changes in the mortality profile, under-registration of death counts, and ill-defined causes of death. Cad Saude Publica. 2014; 30(8):1721-1730.

Synthetic Extinct Generations Adjusted (adjusted SEG) or adjusted or hybrid Extinct Generation method

Hill, You, and Choi in 2009 proposed that the combination of the methods of Hill (1987) and Bennett and Horiuchi can be more robust than the application of the two methods separately. From this, they define the adjusted SEG method, which consists of applying the GGB method to obtain estimates of the change in census coverage (k1/k2) and then using this estimate to adjust one of the two demographic censuses (population enumeration). Then, we applied the SEG method of Bennett and Horiuchi and used the adjusted population to obtain the degree of coverage of the mortality data.

The present analysis applied the SEG method adjusted for total Brazil according to sex. Figure 2 shows a trend of better coverage at younger ages and lower coverage at older ages. Similar to the visual inspection of the GGB method (Figure 1), the curves with the coverages exclude the age group from 0 to 4 years and the open range of 80 years and over. Generally, we believe this is due to better information accuracy in young adults. The SEG method estimated a degree of coverage c = 98% for men and 96% for women. Already the SEG-adjusted average coverage becomes 96% for men and 93% for women.

Regarding applying the three different methods, we observed that the results are relatively similar, all with a degree of coverage above 90% for both sexes. The three methods required as input are the age distributions of the population from two censuses and the deaths recorded by age. Ultimately, they all provide a correction factor that we multiplied by the observed adult mortality rates to obtain the corrected adult mortality rates. Since the SEG method estimates the completeness of the death record for the two census years, it considers the cumulative effect of growth rates and deaths. Because of this, this method seems to be less sensitive to an eventual coverage differential by age group, unlike the GGB method[12].

On the other hand, the GGB and adjusted SEG methods estimate the completeness of census year two concerning census year 1. Due to the nature of the calculations, the GGB has the advantage of considering changes in coverage between the analysis periods. While the GGB method estimates a sub-enumeration of one of the two input populations concerning the other and applies this correction, the SEG method applies a correction to one of the populations through the period growth rate. In this case, the decision of which population is given considering the direction of the growth rate. If there is growth, the final population is adjusted; if there is a decline, it is adjusted to the initial population[13].

We designed the visual inspection of the steps of each method, presented in the previous figures, in simple data sheets. The statistics suggest that the results of the SEG and adjusted SEG methods confirm the analysis presented by the GGB method. To prove this statement, we used the R package DDM (Death Registration Coverage Estimation, version 1.0-0) to obtain accurate estimates. The estimates presented by the routine of the package described were: for males, the GGB showed coverage of 100.1%, SEG 97.4%, and adjusted SEG 93.6%; for females, the GGB obtained coverage of 98.4%, the SEG 96.2%, and the adjusted SEG 91.9%. Finally, we choose the adjusted SEG method. It is important to

---

[12] ADAIR T, LOPEZ AD. Estimating the completeness of death registration: An empirical method. PLoS One. 2018; 13(5):e0197047.

[13] MURRAY CJ, RAJARATNAM JK, MARCUS J, LAAKSO T, LOPES AD. What can we conclude from death registration? Improved methods for evaluating completeness. PLoS Med. 2010; 7(4):e1000262.

emphasize that although the demographic methods used previously are used for data obtained in the demographic censuses, we performed the analysis with data from the Mortality Information System through DATASUS (SIM/DATASUS). Generally, this information system's quality of mortality data is suitable for Brazil. Thus, we applied the correction factor calculated from the degree of underreporting of deaths in the years of study.

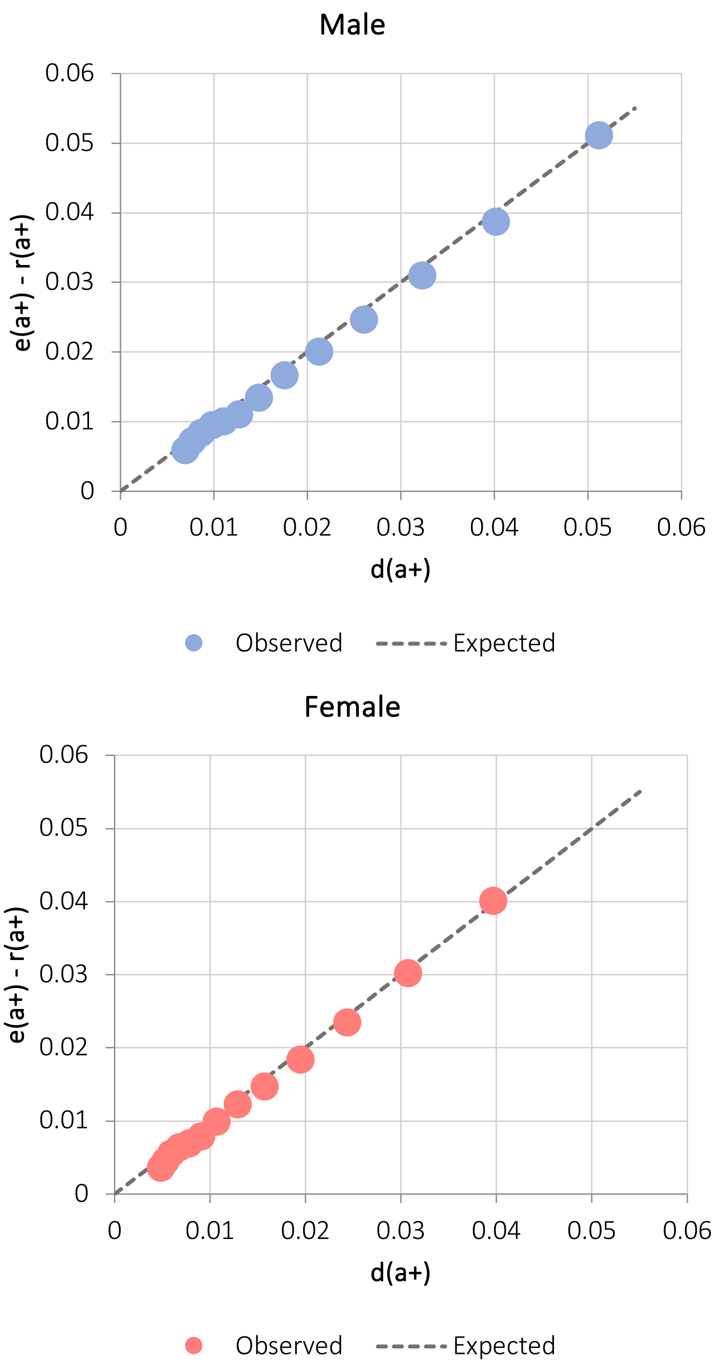

Figure 1: Diagnosis of the difference between partial-birth rate and annual growth vs. death rate using the General Growth Balance (GGB) method. Brazil, 2000 and 2010.
Source: Mortality Information System (SIM/DATASUS), 2000 and 2010.

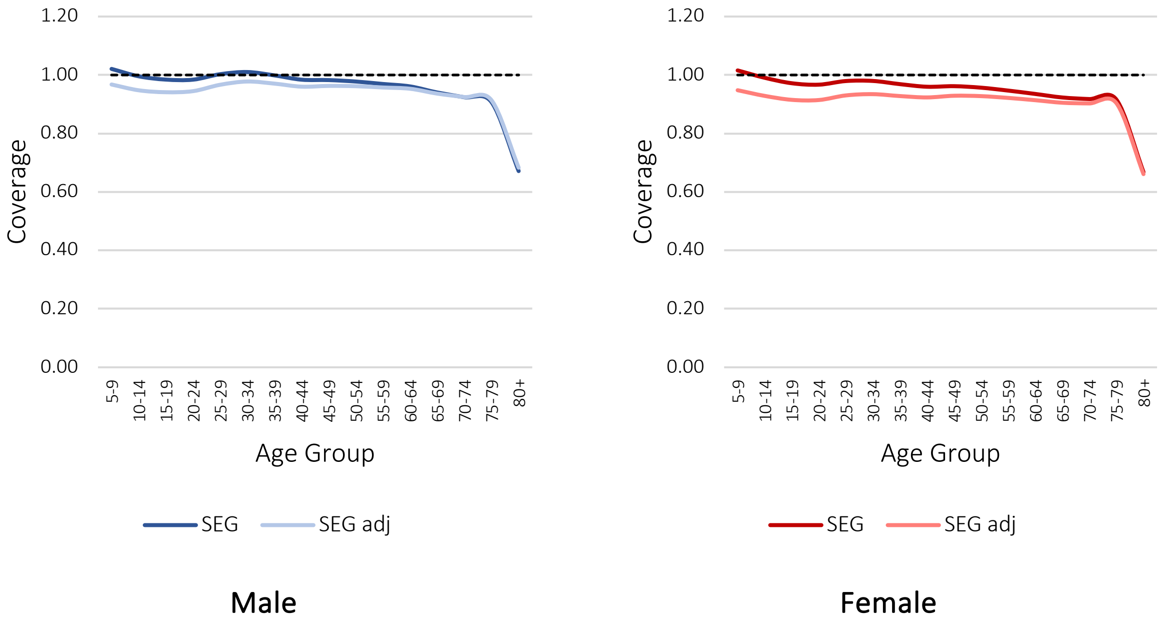

Figure 2: Age coverage of deaths by sex according to correction method. Brazil, 2000 and 2010.
Source: Brazilian Bureau of Statistics (IBGE), Census 2000 and 2010Mortality Information System (SIM), Ministry of Health.

Correction of causes of death

Although the coverage of information on deaths in Brazil has been adequate, another aspect related to record quality is information according to the cause of death. Indeed, erroneous estimates of mortality rates are a long-standing concern affecting the study of cause-specific mortality in epidemiology and demography. The absence of a defined cause in death records also differs according to specific characteristics, such as sex, race/skin color, and age.

The number of deaths with ill-defined or unreported causes has been falling in the country. In 2003, there was a proportion close to 4%, which dropped to below 3% until 2015, with a slight increase, up to 3.3% in 2018. However, we observed a more significant proportion among men, and the discrepancy has been increasing over time (relative difference of 2.76% in 2003 and 17.47% in 2018). Concerning race/skin color, there is a systematically higher proportion of ill-defined causes/no information among blacks, with an average difference of 41% (range in the period 37.89% – 46.75%). Finally, about age groups, the intermediate age groups (30 to 39 years old, 40 to 49 years old, and 50 to 59 years old remain with more significant proportions than the other groups. These groups, it is worth mentioning, are the groups in which, hypothetically, deaths from desperation are more prominent. That said, we chose to carry out the distribution.

We redistributed these deaths by year, sex, age group, and race/skin color for the ICD 10 groups and then to the specific causes within each group. This step aimed at correcting the proportion of deaths classified as ill-defined causes (ICD 10 – R99),

We performed this method in three stages, according to the methodology proposed by the World Health Organization (WHO) (Mathers et al., 2003):

- Step 1: we verified the proportion of deaths by each cause group relative to the total number of deaths;
- Stage 2: we multiplied these proportions by the number of deaths classified as undetermined or ill-defined causes per year, age group, sex, and race/skin color;
- Stage 3: we added the value obtained in the previous stage to the number of original deaths in each group of causes, thus getting the number of corrected deaths.

These three steps are then repeated within each group for redistribution by specific causes within each group, considering the codes of causes associated with despair cited in the methods. We then applied the correction factor to the number of deaths and redistributed the deaths by cause. In this way, we obtained data adjusted for the level of mortality and equally to correct for the pattern by a specific cause. These procedures guarantee the necessary adjustments to estimate the mortality rate based on the correction of the numerator of the rates. On the other hand, to ensure equal quality of information on the population (therefore, on the denominator of the fraction), we used population estimates made by the Brazilian Institute of Geography and Statistics (IBGE).
